# Identification of core genes associated with different phosphorus levels in quinoa seedlings by weighted gene co-expression network analysis

**DOI:** 10.1186/s12864-023-09507-x

**Published:** 2023-07-15

**Authors:** Shan Zhang, Jian Liu, Lian Shi, Qianchao Wang, Ping Zhang, Hongxin Wang, Junna Liu, Hanxue Li, Li Li, Xinyi Li, Liubin Huang, Peng Qin

**Affiliations:** 1 College of Agronomy and Biotechnology, Yunnan Agricultural University, Kunming, 650201 China; 2 Institute of Agricultural Sciences of the Lixiache District, Yangzhou, 225007 China; 3 Yuxi Academy of Agricultural Sciences, Yuxi, 653100 China

**Keywords:** Weighted gene co-expression network analysis, Quinoa, Phosphorus levels, Transcription factors

## Abstract

**Background:**

Quinoa is a highly nutritious and novel crop that is resistant to various abiotic stresses. However, its growth and development is restricted due to its limited utilization of soil phosphorus. Studies on the levels of phosphorus in quinoa seedlings are limited; therefore, we analyzed transcriptome data from quinoa seedlings treated with different concentrations of phosphorus.

**Results:**

To identify core genes involved in responding to various phosphorus levels, the weighted gene co-expression network analysis method was applied. From the 12,085 expressed genes, an analysis of the gene co-expression network was done. dividing the expressed genes into a total of twenty-five different modules out of which two modules were strongly correlated with phosphorus levels. Subsequently we identified five core genes that correlated strongly either positively or negatively with the phosphorus levels. Gene ontology and assessments of the Kyoto Encyclopedia of Genes and Genomes have uncovered important biological processes and metabolic pathways that are involved in the phosphorus level response.

**Conclusions:**

We discovered crucial new core genes that encode proteins from various transcription factor families, such as MYB, WRKY, and ERF, which are crucial for abiotic stress resistance. This new library of candidate genes associated with the phosphorus level responses in quinoa seedlings will help in breeding varieties that are tolerant to phosphorus levels.

**Supplementary Information:**

The online version contains supplementary material available at 10.1186/s12864-023-09507-x.

## Background

Quinoa is an annual dicotyledonous herb belonging to the family Amaranthaceae [1]. It is an important pseudo-cereal as it is environmentally resistant and highly nutritive [2]. Approximately 7000 years ago, it was domesticated for the first time in the Andean countries of South America [3], with large-scale biological diversity [4]. There are approximately 250 quinoa species worldwide [2]. In a report from the United Nations' Food and Agriculture Organization (FAO), quinoa grains provide all essential amino acids and meet the dietary requirements established for human nutrition [5]. Quinoa grains are the main edible part that are gluten-free and are rich in proteins, essential amino acids, essential minerals, and vitamins. It has a high nutritional value for humans and animals. The total protein, fat, and dietary fiber content of fresh quinoa grains per 100 g is 9.1–15.7 g, 4.0–7.6 g, and 8.8–14.1 g, respectively [6]. Because of these nutritional characteristics and health benefits, quinoa is considered a novel health food and sometimes as "super food" [2].

A crucial component for plant development and growth is phosphorus [7]. Phosphorus is the second most limiting nutrient after nitrogen in terrestrial and aquatic ecosystems and the most inaccessible nutrient required by plants [8]. Plants contain up to 0.5% phosphorus by dry weight, where it participates in several plant functions like photosynthesis, respiration, energy generation, nucleic acid biosynthesis, and as a constituent of various plant structures like phospholipids [9]. Phosphorus is the most challenging macronutrient to get while being essential for plant development and metabolism due to its limited availability and poor recovery from applied fertilizers in the majority of agricultural soils [10]. Global phosphorus reserves are being depleted at an even higher rate and it is expected that by 2050 there may not be any reserves of soil phosphorus left. This poses a potential threat to sustainable crop production. The soil is likely to absorb the bulk of fertilizer-applied phosphorus, rendering it unavailable to plants without certain adaptations. However, available soil phosphorus and crop yields can be increased by applying phosphorus-containing fertilizers for sustainability [11]. Therefore, research to discover the core genes of crops under phosphorus levels is necessary to improve crop quality and enhance crop adaptation.

Weighted gene co expression network analysis (WGCNA) constructs a gene co expression network using multiple sample transcriptome datasets that can be used to mine core genes in the gene co-expression network and to investigate the biological relationships between co-expressed gene modules associated with target traits [12]. In plant studies, WGCNA has been employed frequently. Wu et al. identified four modules significantly related to smut resistance by using WGCNA. The study analyzed 36 samples transcriptome data of two sugarcane types, screened 38 candidate hub genes, to comprehend the molecular basis of sugarcane smut resistance in greater detail, and provided new genetic resources for sugarcane smut resistance breeding [13]. Wang et al. carried out WGCNA analysis on two varieties of rice at seven time points under high temperature levels, and identified four modules related to the heat level, and extracted the modules' core genes [14]. Zhu et al. carried out WGCNA analysis on three transcriptomic data of rice under salt treatment and control conditions and identified three modules highly related to salt response. The study also identified important new central genes encoding different family of proteins, they include transcription factors and PP2-13, LEA4-5, CHL27, CAM, DUF630/632, DUF581, PP2-13, DUF581 [15],

In this study, WGCNA analysis was carried out on quinoa seedlings under different phosphorus treatment conditions, and two modules under low- and high-phosphorus treatment conditions were obtained. Through the prediction of the KME value and transcription factors of the different genes in the modules, five core genes of the modules were determined by the two modules. These core genes were MYB transcription factor family including the gene-LOC11722828, gene- LOC110718502, and gene-LOC110724431, ERF transcription factor family including the gene-LOC110688029 and gene-LOC110687923, WRKY transcription factor family including the gene-LOC110707263, etc. Several transcription factor families are recognized to play a significant protective effect in plants in response to phosphorus level circumstances from the literature that is currently accessible. The core genes screened in this study provide some guidance for the future breeding of quinoa under phosphorus level conditions, and these genes can be further exploited and validated for model plant functions.

## Results and analysis

### Construction of quinoa weighted co-expression network

**Fig. 1:**
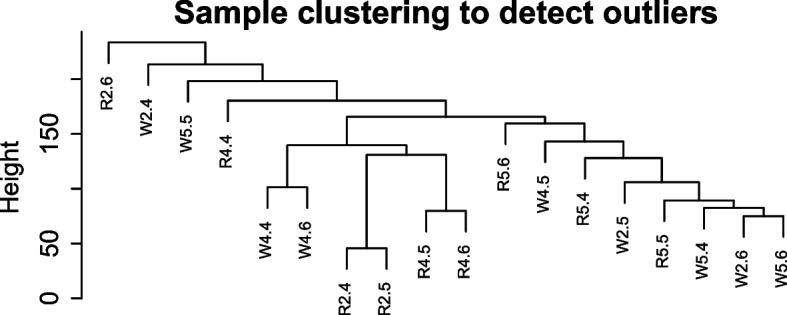
Clustering diagram of quinoa seedling samples. The horizontal coordinates represent sample clustering, and one column represents one sample. The clustering is based on the similarity of gene expression between samples, and the closer the gene expression between samples, the closer they are to each other (R represents red quinoa (Dianli-1299) and W represents white quinoa (Dianli-71); W2/R2 represents a phosphorus treatment level of 112.5 kg/hm^2^;W4/R4 represents a phosphorus treatment level of 0 kg/hm^2^;W5/R5 represents a phosphorus treatment level of 337.5 kg/hm^2^)

By eliminating genes with low expression, a total of 12,085 genes were employed to build a weighted gene co-expression network. Cluster analysis was carried out on 18 samples to check the Outlier of sample clustering, and the results showed that there were no outlier (Fig. 1), Among the 18 samples, R represents red quinoa and W represents white quinoa; 2 represents a gradient of 112.5 kg/hm^2^ for P_2_O_5_ treatment, and 4 represents a gradient of 0 kg/hm^2^ for P_2_O_5_ treatment; 5 represents a P_2_O_5_ treatment gradient of 337.5 kg/hm^2^, with each treatment undergoing three biological and technical replicates. They are R2.4, R2.5, R2.6 and W2.4, W2.5, W2.6, respectively. R4.4 R4.5 R4.6 and W4.4 W4.5 W4.6. R5.4 R5.5 R5.6 and W5.4 W5.5 W5.6.

**Fig. 2:**
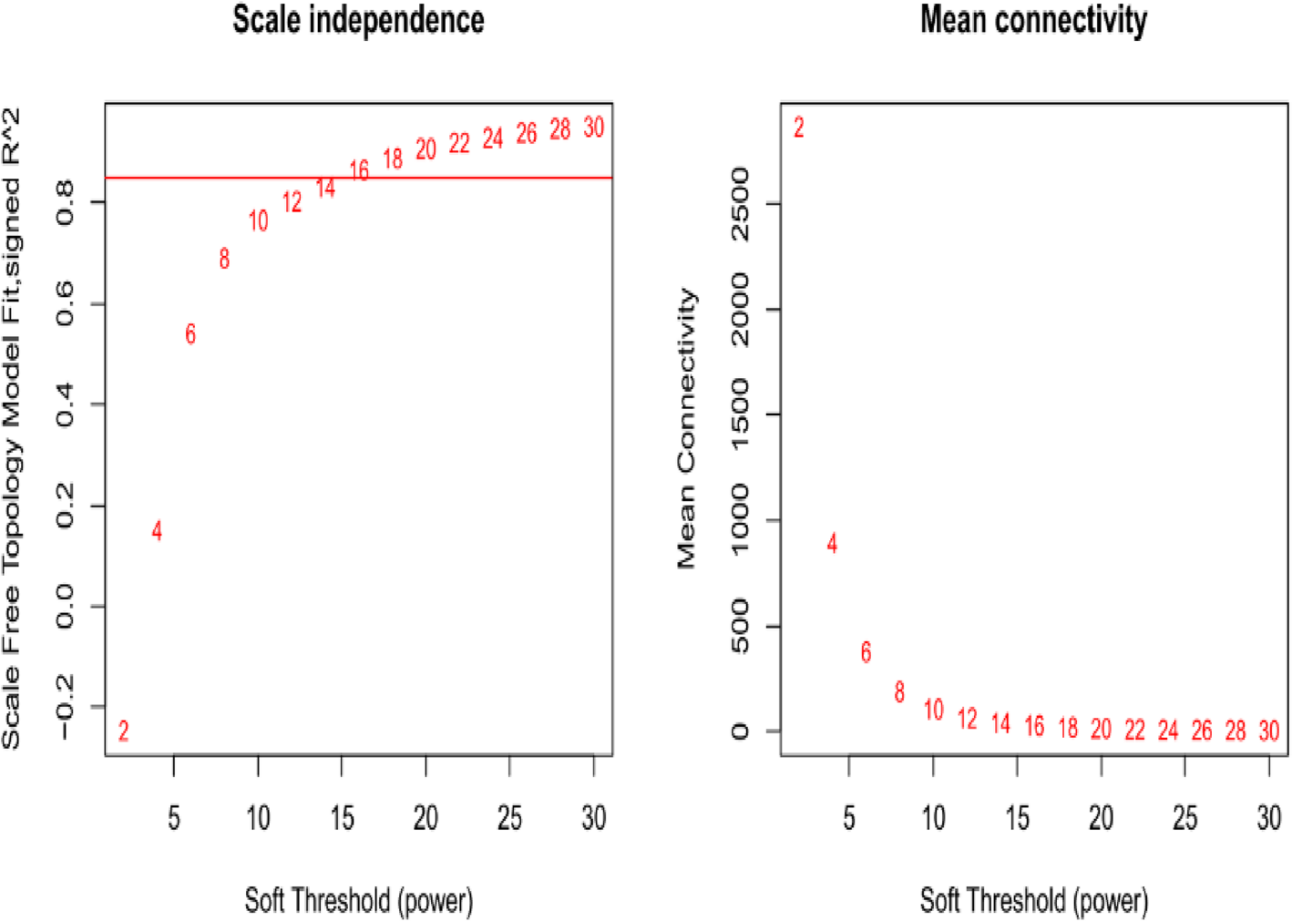
Network topology of quinoa seedlings with different soft threshold powers. x-axis indicates the weight parameter β. y-axis in the left panel indicates the square of the correlation coefficient between log(k) and log(p(k)) in the corresponding network. The y-axis of the right panel represents the average of all gene adjacency functions in the corresponding gene module. The approximate scale-free topology is obtained at a soft threshold power of 16 for both genotypes

**Fig. 3:**
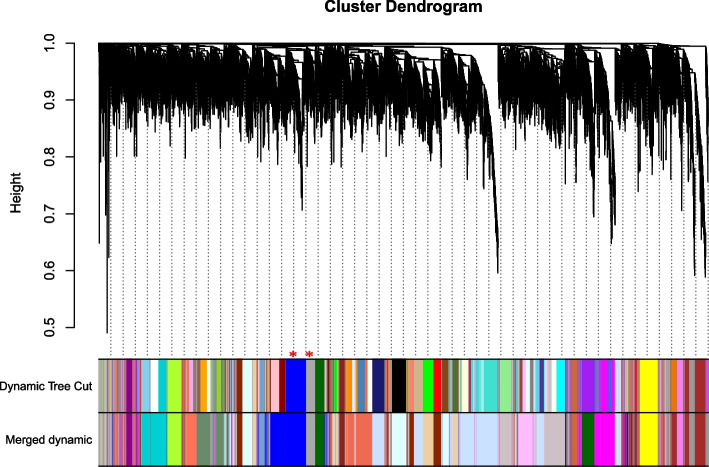
Gene modules identified by weighted gene co-expression network analysis (WGCNA) of phosphorus levels in quinoa seedlings. Gene tree maps obtained by color clustering dissimilarity of the corresponding modules based on consistent topological overlap and color row indications. Each row represents a color-coded module containing a set of highly linked genes

Subsequently, Based on the requirement that the square of the correlation coefficient was near to 0.85 and a specific level of gene connection was required, the PickSoftThreshold function was used to choose the proper weighting coefficient. The co-expression network was built with a -value of 16 (Fig. 2). After this dynamic cut method was used to divide the network modules, small modules with high similarity were combined, and finally twenty-five modules were constructed (Fig. 3). The expression of genes in the module may be highly correlated with different phosphorus levels under treatment conditions.

### Identification of specific modules related to phosphorus levels in quinoa

**Fig. 4:**
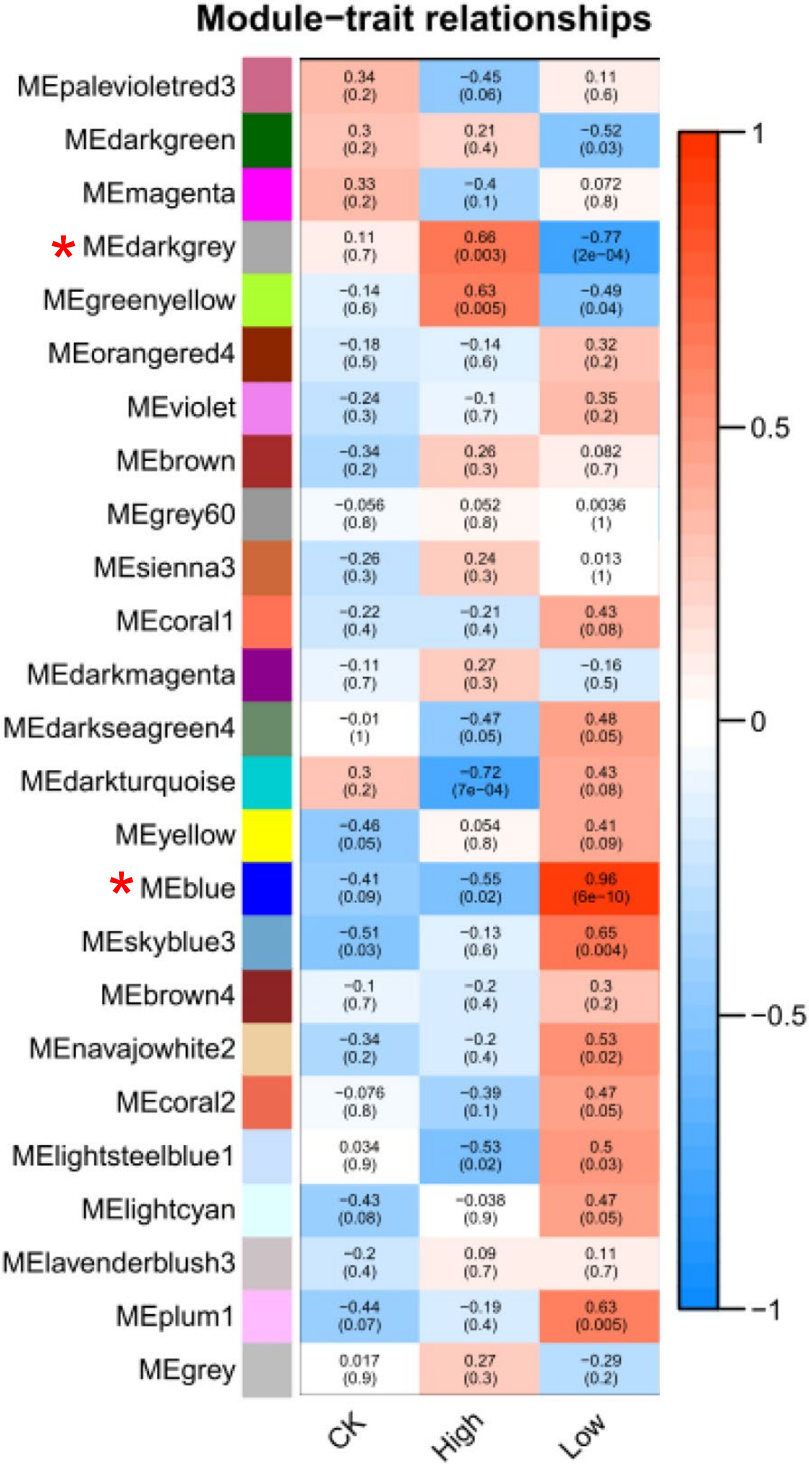
Heat map of quinoa seedling gene co-expression network modules associated with different phosphorus levels. Each row in the table corresponds to a consensus module and each column corresponds to a time point. Module names are shown on the y-axis and time points are shown on the x-axis. The table is color-coded by correlation according to the color legend. The strength and direction of the correlations are shown on the right side of the heat map (red, positive correlation; blue, negative correlation)

For each module, gene co-expression was summarized by the signature gene (i.e., the first component expressed of the gene belonging to the module), and the correlation between each signature gene and sample treatment conditions, such as low phosphorus and high phosphorus, was calculated (Fig. 4). Based on the correlation between modules and sample treatments, with correlation > 0.5 and *P* < *0.05*, two gene co-expression modules specifically associated with phosphorus levels were identified in this experimental study. Dark-grey module was positively correlated with high phosphorus(*r* = 0.66, *p* = 0.0003) treatment conditions and negatively correlated with low phosphorus(*r* = -0.77, *p* = 2e-04) treatment conditions. these two modules will be used as specific modules related to phosphorus levels for in-depth research, exploring the core genes in the modules. Subsequently, five core genes were identified through analysis in this module: gene-LOC110707263, gene-LOC110687923, gene-LOC110718502, gene-LOC110724431, and gene-LOC110726774.While as the blue module was negatively correlated with high phosphorus(*r* = -0.55, *p* = 0.02) treatment conditions and positively correlated with low phosphorus(*r* = 0.96, *p* = 6e-10) treatment conditions. Five core genes were screened in this module: gene-LOC110688029, gene-LOC11722828, gene-LOC110687734, gene-LOC110706317, gene-LOC110723306.

### Analysis of quinoa phosphorus levels related to specific modules GO and KEGG enrichment

**Fig. 5:**
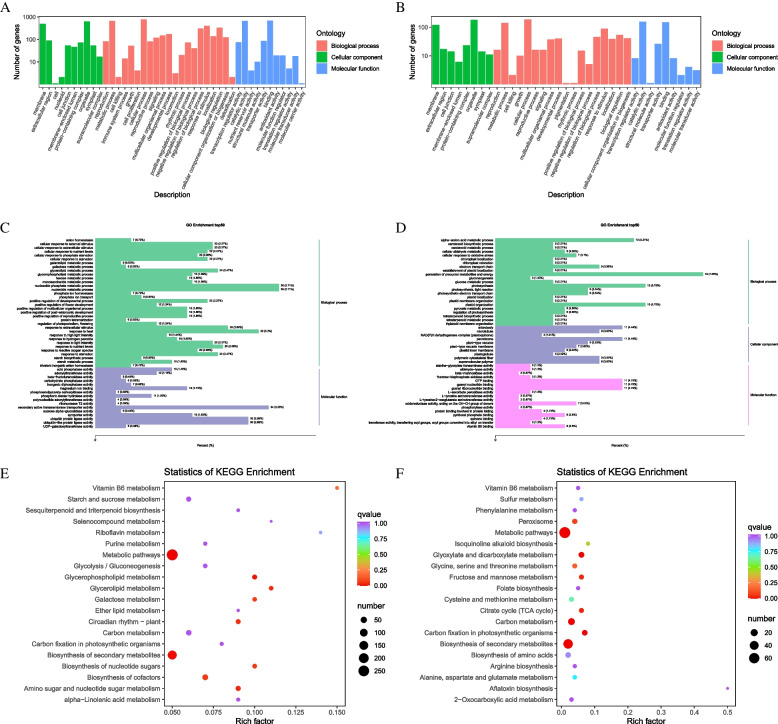
GO annotation (**A**, **B**), GO enrichment (**C**, **D**) and KEGG enrichment (**E**, **F**) analysis of candidate genes for BLUE (**A**, **C**, **E**) and Dark-grey (**B**, **D**, **F**) modules. x-axis of GO annotation shows GO terms for BP, CC and MF. y-axis shows the number of genes associated with GO terms. x-axis of GO enrichment shows the percentage of genes in GO terms and y-axis shows the percentage of genes in KEGG terms. The y-axis shows GO enrichment terms. x-axis for KEGG enrichment shows the percentage of genes in KEGG terms, and the y-axis shows KEGG enrichment terms

To explore the functional classification and metabolic pathways of genes in which genes responsive to different phosphorus levels are involved, we conducted GO analysis on the top 50 genes in the Blue and Darkgrey modules, and the results are divided into three categories (Fig. 5A, B): biological processes, molecular functions and cellular components. The Blue module (Fig. 5C) enriched 1690 biological processes, 658 molecular functions, and 199 cellular components, mainly in nucleoside phosphate metabolic process(GO:0006753)**,** response to heat(GO:0009408), secondary active transmembrane transporter activity(GO:0015291), and ubiquitin protein ligase activity(GO:0061630. This indicates that low phosphorus treatment may affect the growth and development of quinoa seedlings by affecting signal transduction pathways and transport protein activity. The Dark-grey module (Fig. 5D) enriched to 999 biological processes, 305 molecular functions, and 138 cell components, mainly concentrated in the generation of precursor metabolites and energy(GO:0006091), photosynthesis(GO:0015979), and plastid organization(GO:0009657), microbody(GO:0042579), peroxisome(GO:0005777), GTP binding(GO:0005525), and guanyl nucleotide binding(GO:0019001) (Table S1). This indicates that high phosphorus treatment may mainly affect the growth and development of quinoa seedlings by affecting their photosynthesis. The analysis of GO enrichment results indicates that in the co expression module constructed by WGCNA, the genes of the Blue and Darkgrey modules play important roles in the growth of quinoa seedlings under different phosphorus treatment levels.

Annotating two specific module genes highly correlated with different phosphorus levels into the KEGG database can provide a more intuitive understanding of the main functions of the module genes. KEGG enrichment analysis of the genes in the specificity module revealed that the blue module (Fig. 5E) was involved in glycerophospholipid metabolism(ko00564), glycerol-lipid metabolism(ko00561), galactose metabolism(ko00052), Amino acid and nucleotide sugar metabolism (ko00520), and sugar nucleotide biosynthesis(ko01250), The dark-grey module (Fig. 5F) can be enriched in glyoxylate and dicarboxylate metabolism(ko00630), fructose and mannose metabolism(ko00051), tricarboxylic acid cycle(ko00020), carbon metabolism(ko01200), glycine serine and threonine metabolism(ko00260). The analysis results indicate that these modular genes may play a key role in the response of quinoa seedlings to different phosphorus levels through amino acid, energy, and sugar metabolism pathways.

### Identification of core genes in the significant co-expression module of quinoa phosphorus levels and construction of gene interaction network

**Fig. 6:**
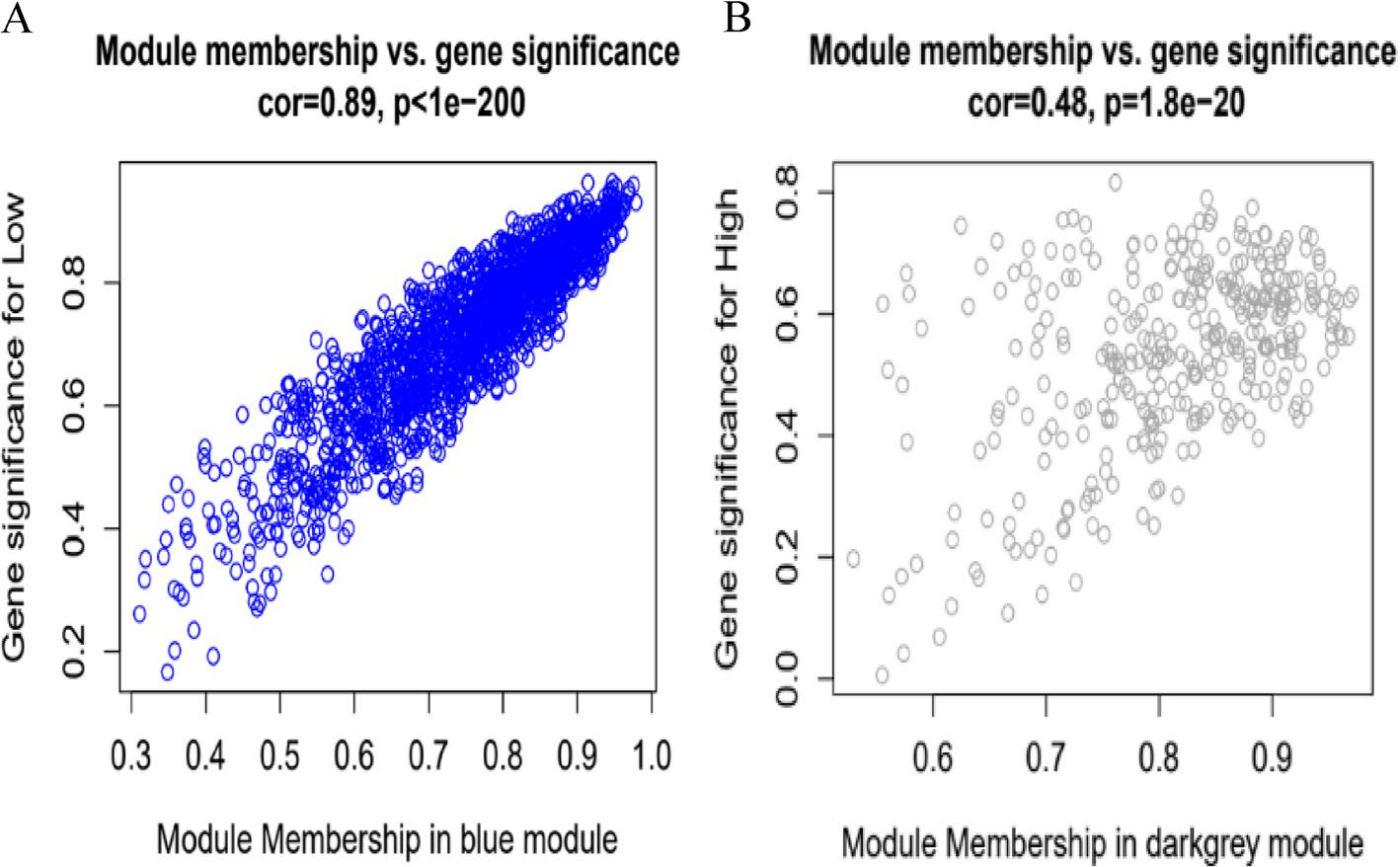
Scatter plot of gene importance (GS) versus module membership (MM) in Blue (**A**) and Dark-grey (**B**) modules. In both modules, there is a significant correlation between GS and MM

Based on the requirement that the square of the correlation coefficient was near to 0.85, the PickSoftThreshold function was used to choose the proper weighting coefficient. The co-expression network was built with a -value of 16. Considering that the Blue and Darkgrey modules have a relatively high correlation with high and low phosphorus levels, they may have potential genes that respond to high and low phosphorus levels. Therefore, these two modules are used to construct a gene interaction network. Scatter plots of GS and MM values for the blue (Fig. 6A) and dark-gray (Fig. 6B) modules were plotted. Key genes were screened by setting GS (Gene Significance) to > 0.4 and |MM(Module Membership) > 0.8. We obtained 1692 and 331 pivotal genes in the two modules, respectively (Table S2).

**Fig. 7:**
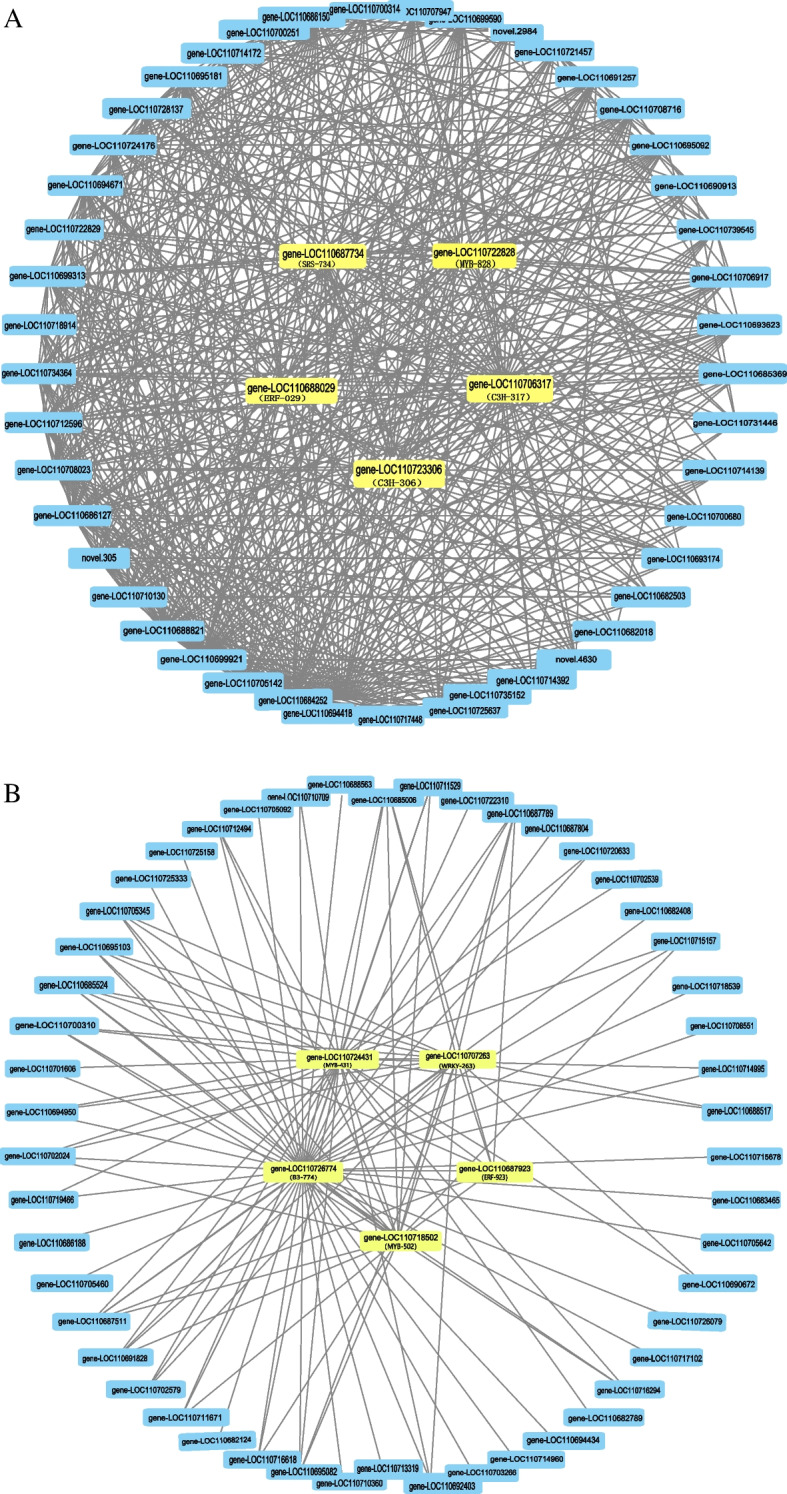
Candidate core genes of Blue (**A**) and Dark-grey (**B**) obtained from the interaction network analysis with known core genes. The yellow color represents the core genes

Genes with KME values in the top 20 were selected as the initial candidate genes (Table S3). Subsequently, the betweenness (BC) values of the candidate genes were calculated using the cytoNCA plug-in in cytoscape 3.9.1 software to screen the core genes. Five core genes were identified for each module. The core genes identified in the blue module (Fig. 7A) were: gene-LOC110688029, gene-LOC11722828, gene-LOC110687734, gene-LOC110706317, gene-LOC110723306. The dark-gray module (Fig. 7B) core genes are: gene-LOC110707263, gene-LOC110687923, gene-LOC110718502, gene-LOC110724431, gene-LOC110726774.

The protein sequence comparison of the module hub gene through the plant TFDB website revealed that the core genes in the dark-grey module were better than those in the MYB, WRKY, ERF, and B3 transcription factor families, respectively. The top five core genes in the blue module were mostly better than those of the MYB, ERF, C3H, and SRS transcription factor families, and the functions of the focal candidate core genes were further understood by annotation into the Tair website (Table 1).

**Table 1:** Functional annotation of core genes in the phosphorus level-related specificity module

Modules	Module candidate hub genes	Transcription Factor Family	Gene function
Blue	gene-LOC110688029 (ERF-029)	ERF family protein	Linoleic acid 9S-lipoxygenase-4 protein, a senescence-associated protein whose expression is regulated by cytokinin
	gene-LOC11722828 (MYB-828)	MYB family protein	Encodes a polygalacturonase inhibitory protein involved in defense responses
	gene-LOC110687734 (SRS-734)	SRS family protein	Encoding PAP15, a violet acid phosphatase with phytase activity, PAP15 may mobilize phosphorus reserves in plants, especially during seed and pollen germination
	gene-LOC110706317 (C3H-317)	C3H family protein	Encodes a member of the T2 family of ribonucleases that responds to inorganic phosphate starvation and inhibits anthocyanin production. Its expression is responsive to both phosphate and phosphite in the root system
	gene-LOC110723306 (C3H-306)	C3H family protein	Encodes an acid phosphatase involved in plant adaptation to Pi deprivation
Dark-grey	gene-LOC110707263 (WRKY-263)	WRKY family protein	Encodes SVR3, a putative chloroplast TypA translational elongation GTPase. SVR3 is essential for plants to develop functional chloroplasts in response to cooling stress (8 °C)
	gene-LOC110687923 (ERF-923)	ERF family protein	Encoding HCF173, the protein HCF173 is located in chloroplasts, where it is primarily associated with the membrane system and is part of a higher molecular weight complex in which psbA mRNA is a component of the complex
	gene-LOC110718502 (MYB-502)	MYB family protein	Protein chaperone-like protein of POR1-like
	gene-LOC110724431 (MYB-431)	MYB family protein	Encodes a vesicular glucose export factor that is induced in response to factors that activate the vesicular glucose pool (e.g., darkness, heat stress, and injury) and is inhibited under conditions that trigger glucose accumulation in the vesicles (e.g., cold stress and external sugar supply)
	gene-LOC110726774 (B3-774)	B3 family protein	Encoding HCF173, the protein HCF173 is located in chloroplasts, where it is primarily associated with the membrane system and is part of a higher molecular weight complex in which psbA mRNA is a component of the complex

## Discussion

One of the macronutrients that is crucial for the growth and development of plants is phosphorus. It serves as a crucial structural component for numerous macromolecules, including phospholipids found in biological membranes, high-energy chemicals, and nucleic acids. Phosphorus is also involved in many cellular processes, including energy conservation, carbon assimilation, photosynthesis, respiration, and regulation of many enzymes [16]. Although abundant, soil phosphorus is often limited to plants because of its low bioavailability [17]. Phosphorus levels can limit crop growth and reduce crop yield, and can lead to significant reductions in maize, rice, or other crop yields [18–20], Farmers apply large amounts of phosphorus fertilizer to obtain high yield. Unfortunately, excessive phosphorus application not only raises the input–output ratio but also causes environmental pollution and the buildup of toxic substances in the soil. [21], Cong et al. [22] used a diverse mixed cropping system by growing phosphorus-efficient genotypes to improve the phosphorus use efficiency of crops while mitigating the negative environmental impacts. This approach can conserve non-renewable phosphorus resources and improve the nutritional quality of food. Therefore, understanding the core genes of crops under varying phosphorus levels, improving phosphorus use efficiency, and breeding quinoa genotypes with high phosphorus absorption efficiency are important means to address these issues.

Plants develop their own molecular mechanisms to cope with varying phosphorus levels in order to adapt to the environmental conditions. Low phosphorus levels can inhibit plant growth and affect plant morphology, resulting in slow aboveground plant growth and a well-developed root system [23].One of the responses of plants to low exogenous phosphorus conditions is to change the root conformation. For example, primary root length is decreased in Arabidopsis plantlets cultivated under low phosphorus circumstances, whereas lateral root growth is promoted [24], which enhances crop respiration, leading to high carbohydrate consumption, green leaf color, thick and dense leaves, early development of reproductive organs, and slowed stem and leaf growth, thus causing premature plant failure. High phosphorus levels also encourage root growth and increase the quantity of roots, producing short and dense roots. Crop productivity and quality are reduced as a result of the changing ratio of aboveground to root growth [23]. This agrees with the findings of the current investigation. In the present study, quinoa seedlings grown for up to 30 days in the low phosphorus treatment started to show stiffness, whereas the high phosphorus treatment conditions and the control could continue their growth. In addition, plant height and leaf area were minimal in the low phosphorus treatment condition, whereas quinoa seedlings in the high phosphorus treatment condition grew faster than the control group but did not cause early plant failure because the gradient set in the high phosphorus group was not high enough.

There are also many effects of different phosphorus conditions on crops. Xu W et al. [25] found that under phosphorus deficiency conditions, white feather fan beans may participate in auxin regulation through LaABCG36s and LaABCG37s, promote the formation of cluster roots, and improve plant tolerance to low phosphorus; Wang Y et al. [26] found that after about a month of low phosphorus stress, the content of citric acid and malic acid in the roots of oats was significantly upregulated, and 48 related genes were significantly upregulated; Tingting Sun et al. [27] found that different phosphorus conditions have a certain impact on apple roots and leaves, and their phenotypic characteristics, flavonoid and anthocyanin content have a certain impact. In this study, we have a preliminary understanding of the function of differential genes under different treatment conditions. Under low phosphorus conditions, they are mainly enriched in glycerol phospholipid metabolism, glycerol ester metabolism, galactose metabolism, sugar nucleotide biosynthesis, amino acid and nucleotide sugar metabolism. Under high phosphorus conditions, it can be enriched in acetaldehyde and dicarboxylate metabolism, glycine serine and threonine metabolism, fructose mannose metabolism, tricarboxylic acid cycle, and carbon metabolism.

Subsequently, the two modules were screened for core genes by calculating the KME value. Twenty candidate genes were initially screened for each module, and BC value accounting was carried out using Cytoscape software. Five core genes were identified for each module, and gene function annotation and transcription factor prediction were performed. Through this prediction, we observed that most of the core genes were compared with the WRKY transcription factor family, ERF transcription factor family, MYB transcription factor family, and C3H transcription factor family. Transcription factors bind specifically to a cis-acting area in the promoter of a target gene, which allows them to play a significant role in gene production. and regulating the expression of downstream genes as trans-acting elements [28]. The degree to which plants respond to abiotic stress is significantly influenced by transcription factors. Under quinoa phosphorus treatment conditions, most of the core genes were annotated in the WRKY, C3H, ERF, and MYB transcription factor families, based on the analysis of sequence comparison results. The core genes gene-LOC11722828, gene-LOC110718502, and gene-LOC110724431 belong to the MYB transcription factor family. Das et al. [29] reported that PHR2 is a major transcriptional regulator of the phosphorus starvation response in rice, which also regulates mycorrhizal roots. PHR2 is a member of the MYB transcription factor family involved in the plant phosphate starvation response, and the colonization of mycorrhizal roots in rice phr2 mutants which was significantly reduced under low phosphorus conditions, whereas ectopic expression of PHR2 promoted colonization of mycorrhizal roots in both low-, medium-, and high-phosphorus environments, suggesting that PHR2 promotes symbiosis of tufts of mycorrhizae under phosphorus starvation conditions, and its inactivation may be responsible for mycorrhizal deficiency under high-phosphorus conditions. The core gene gene-LOC110707263 belongs to the WRKY transcription factor family. Liu [30] et al. found that the soybean GmWRKY46 gene belonging to the WRKY TF family group III is involved in the regulation of soybean P deficiency tolerance. GmWRKY46 was significantly more expressed in low phosphorus-sensitive soybean varieties than in phosphorus-tolerant soybean varieties. It is mainly expressed in roots and is strongly induced by phosphorus deprivation. The core gene gene-LOC110688029 and gene-LOC110687923 belong to the ERF transcription factor family. Chen [31] et al. PalERF2, an AP2/ERF transcription factor gene from Pseudomonas albicans, was discovered. PalERF2 overexpression and knockdown altered tolerance to phosphorus deficiency and drought levels, respectively, relative to WT. Subsequent studies showed that PalERF2y binds to the DRE motif and directly regulates the expression of the drought response genes PalRD20 and PalSAG113y as well as the PSI genes PalPHL2 and PalPHT14. These findings clearly indicate that Populus can recruit PalERF2 to increase Pi uptake and improve drought tolerance. By transcription factor family comparison, the above genes were involved in the protection mechanism of quinoa seedlings against phosphorus level treatment conditions.

Functional annotation of the core genes revealed that quinoa seedlings respond to the harsh environment with low phosphorus levels under low phosphorus conditions mainly by encoding the expression of repressor proteins and acid phosphatases involved in defense reactions. Under weakly acidic (pH 4–7) conditions of the plant body, PAPs can catalyze the hydrolysis of phosphate monoesters and acid anhydrides (such as ATP, ADP, and glycolipids) and release inorganic phosphorus for plant uptake and utilization, thus improving phosphorus utilization by plants [32]. Wang et al. [33]. reported that under conditions of long-term phosphorus deficiency, plant cells induce the expression of intracellular acid phosphatase, which degrades non-essential intracellular organic phosphorus into free inorganic phosphorus to maintain intracellular phosphorus homeostasis. Under high phosphorus conditions, quinoa seedlings adapt mainly by encoding chloroplast-associated proteins. Phosphorus is an important component of chloroplasts, which are the main organ of crop photosynthesis [34], therefore, phosphorus affects the efficacy of photosynthesis, mainly by influencing the number and functional performance of chloroplasts. The functional annotation of these genes revealed further that these key genes are crucial in controlling how quinoa seedlings adjust to their environment's phosphorus levels.

## Conclusion

In summary, a WGCNA-based gene co-expression network was constructed using the quinoa seedlings' transcriptomes under various phosphorus treatment settings. This study's main objective was to screen the core genes of modules based on transcriptomic data using WGCNA analysis. We selected ten candidate pivotal genes by co-expression module under different phosphorus treatment conditions and by KME value calculation analysis, namely gene-LOC110688029(ERF-029), gene-LOC11722828(MYB-828), gene-LOC110687734(SRS-734),gene-LOC110706317(C3H-317),gene-LOC110723306(C3H-306), gene-LOC110707263(WRKY-263), gene-LOC110687923(ERF-923), gene-LOC110718502(MYB-502), gene-LOC110724431(MYB-431), and gene-LOC110726774(B3-774). These potential core genes' transcription factor family study showed that most were from the MYB, WRKY, and ERF transcription factor families, which may play a significant part in how quinoa seedlings react to phosphorus levels. Further studies on the molecular mechanism of the phosphorus level response in quinoa seedlings and the mining of core genes can be performed, laying a solid foundation for further studies on unknown proteins in quinoa and other plants, as well as other abiotic factors.

## Methods

### Material planting and growing conditions

**Fig. 8:**
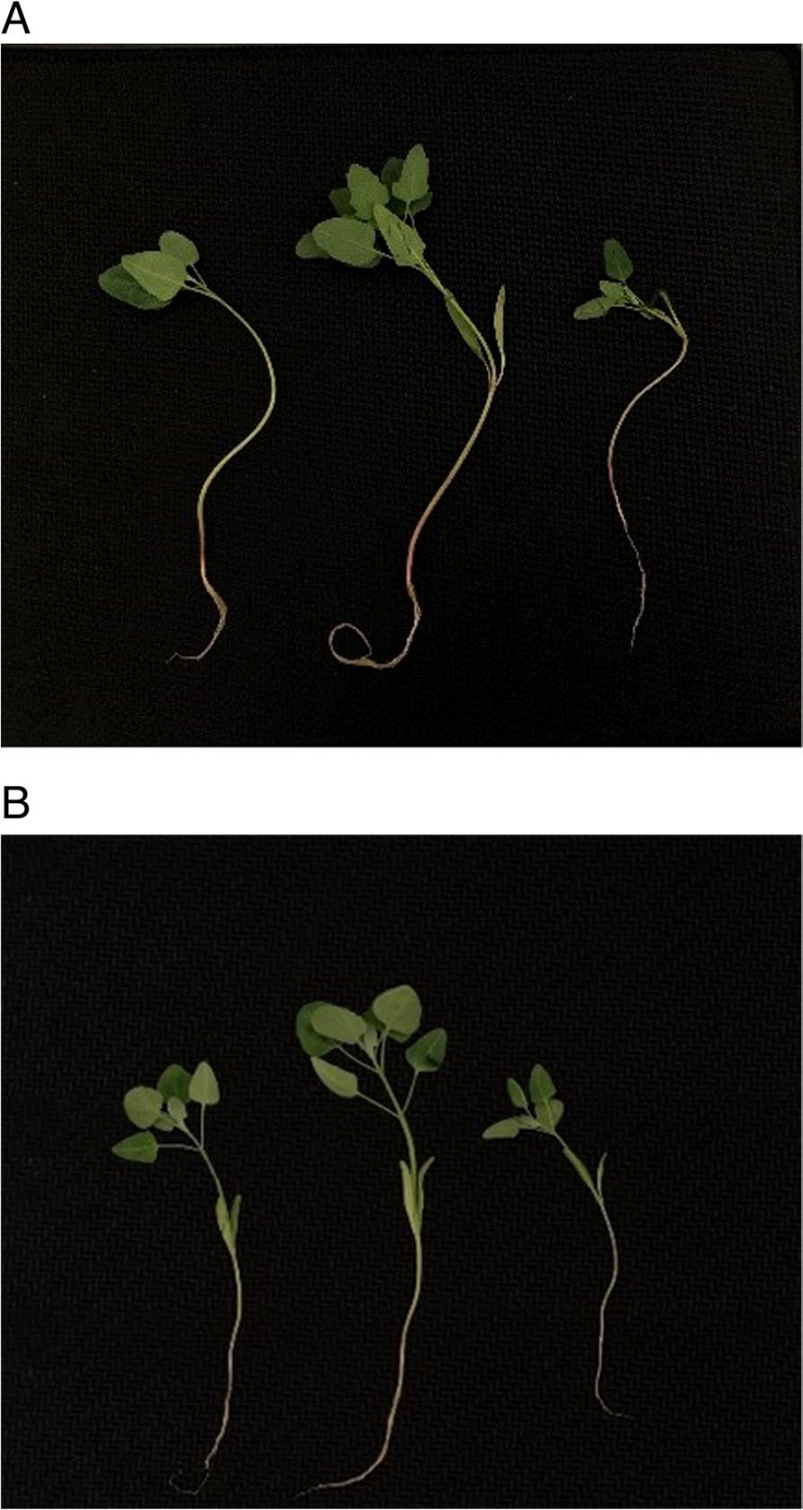
Plant Materials for Quinoa Seedlings, A for red quinoa (Dianli-1299), B for white quinoa (Dianli-71)

Two quinoa types, red quinoa (Dianli-1299, Fig. 8A) and white quinoa (Dianli-71, Fig. 8B), selected by Yunnan Agricultural University and grown at the Yunnan Agricultural University Contemporary Education Research Site (Xundian County, Kunming, Yunnan Province) [35]**,** provided the transcriptomic data used in this study. The seedlings grew in a greenhouse, with an average temperature of 25.6 °C and sunshine duration of approximately 10 h. Fertilizer application was started from the two-leaf stage. The fertilizer consisted of 2.75 g/kg of CH_4_N_2_O, 1.66 g/kg of P_2_O_5_, and 1.18 g/kg of K_2_Og, and the growth of quinoa seedlings under different phosphorus treatments varied 30 d after fertilization. At this point, collect above ground leaf samples of two-leaf stage quinoa seedlings treated with different phosphorus gradients. Having a 25.6 °C average temperature and 0.0 mm of rain on the sampling day; a total of 18 samples were sent to Wuhan Metware Biotechnology Co. Ltd for transcriptome data analysis. In this experiment, we conducted three biological and technical replicates. The sample processing is shown in Table 2, where R represents red quinoa and W represents white quinoa in the analysis; In the data analysis, 2 represents a P_2_O_5_ processing gradient of 112.5 kg/hm2, labeled as the CK group; 4 represents a P_2_O_5_ treatment gradient of 0 kg/hm2, labeled as LP group; 5 represents a P_2_O_5_ treatment gradient of 337.5 kg/hm2, labeled as the HP group; (CK includes R2 and W2; LP includes R4 and W4; HP includes R2 and W2).

**Table 2:** Treatment conditions of quinoa seedling samples

Material name	Material handling (P_2_O_5,_ kg/hm^2^)	Number name	Number callout
Dianli-1299 (Red quinoa)	112.5	R2	CK
	0	R4	LP
	337.5	R5	HP
Dianli-71 (White quinoa)	112.5	W2	CK
	0	W4	LP
	337.5	W5	HP

### Data acquisition

Red quinoa (Dianli-1299) and white quinoa (Dianli-71) were treated with three gradients of phosphorus levels, with P_2_O_5_ gradients of 112.5 kg/hm^2^, 337.5 kg/hm^2^, and 0 kg/hm^2^, respectively. Each gradient treated material underwent three biological replicates, totaling 18 samples. Sent to Wuhan Maitville Biotechnology Co., Ltd. for transcriptome analysis [36].

The experimental process of transcriptome sequencing includes RNA extraction, RNA detection, library construction, and computer sequencing. The RNA extraction process is as follows: 4° C pre cooled centrifuge; Transfer the tissue to a 1.5 ml RNA free EP tube. Place on ice and let stand for 2–3 min. Add 200ul of chloroform to each tube, mix thoroughly, and let stand on ice for 10 min to completely dissociate the nuclear protein complex. Centrifuge at 13,000 rpm for 15 min at 4° C. During this period, take a new EP tube, add 500ul of isopropanol, and pre cool on ice. After centrifugation, transfer the upper aqueous phase (approximately 500 ul) to the new EP tube. Let stand on ice and precipitate with alcohol for 10 min. Centrifuge at 13,000 rpm for 10 min. Remove the supernatant. Wash RNA precipitation once with 1 ml of 75% ethanol. Centrifuge at 12,000 rpm for 5 min. Remove the supernatant and air dry RNA precipitation for 5–10 min. Dissolve RNA in 30-50ul of DEPC treated deionized water. Perform spectrophotometric analysis to determine sample concentration and purity.

The starting RNA for database construction is total RNA, with a total amount of ≥ 1ug. The library building kit used in the library building process is Illumina's NEBNext ® UltraTM RNA Library Prep Kit。After the construction of the library is completed, preliminary quantification is performed using Qubit2.0 Fluorometer, and the library is diluted to 1.5 ng/ul. Then, the insert size of the library is tested using Agilent 2100 bioanalyzer. After the insert size meets expectations, qRT-PCR accurately quantifies the effective concentration of the library (the effective concentration of the library is higher than 2 nM) to ensure the quality of the library. After passing the library inspection, different libraries are pooled according to the effective concentration and target offline data volume requirements, and then used for Illumina sequencing. The basic principle of sequencing is sequencing by synthesis. Add four fluorescent labeled dNTPs, DNA polymerase and connector primers to the sequenced flow cell for amplification. When each sequencing cluster extends the complementary chain, each addition of a fluorescent labeled dNTP can release the corresponding fluorescence. The sequencer captures the fluorescence signal and converts the optical signal into the sequencing peak through computer software, so as to obtain the sequence information of the segment to be tested. Use fastpv0.19.3 to filter offline data, mainly removing reads with adapters; When the N content in any sequencing reads exceeds 10% of the base number of the reads, remove the paired reads; When the low-quality (Q ≤ 20) base number contained in any sequencing reads exceeds 50% of the base number of that read, the paired reads are removed. All subsequent analyses are based on clean reads.

Build an index using HISAT v2.1.0 and compare clean reads with the reference genome (https://www.ncbi.nlm.nih.gov/genome/?Term=Chenopodium+quinoa+Willd) [37] to obtain positional information on the reference genome or gene, as well as sequence characteristic information unique to the sequencing sample, resulting in Mapped Data. New transcript prediction uses StringTiev1.3.4d for new gene prediction. Quantitative gene expression levels were calculated using featureCounts v1.6.2 for gene alignment, and then the FPKM of each gene was calculated based on its length.

### Construction of a weighted gene co-expression network

A gene co-expression network was created using the WGCNA program((version 1.6.1)) in Rstudio (version 4.2.1) [38]. Using the standardized gene expression matrix, a total of 18 sample transcriptome data were used as input data, and the weighted co-expression network was created by screening the top 50% of the genes with the greatest expression variation among the samples using the Genefliter software package in R. PickSoftThreshold was used in the WGCNA package to calculate the weight value and to select a power value of 16. Use blockwiseModules to build a scaleless network, with parameters set by default. The parameters were set to default. WGCNA was used to divide the core genes into 25 modules and calculate the correlation between each module and phosphorus level.

### Identification of phosphorus level-specific modules and functional enrichment analysis of GO and KEGG

Each co-expression module underwent principal component analysis (PCA), with principal component 1 (PC1) being referred to as the eigenvector of that module. To screen the phosphorus-level correlation-specific modules, the correlation coefficients between ME values and different treatments were calculated for each module. The *P*-value is the probability that reflects the likelihood of an event occurring. The *P*-values obtained from the statistical test of significance were generally significant at *P* < *0.05*, and highly significant at *P* < *0.01*. In this study, a module was considered specific if its ME (Module eigengene, ME). Value and trait correlation coefficient |r| were > 0.50 and *P* < *0.03*. The screened specific module genes were used for GO and KEGG enrichment analysis. Specific module genes using KEGG compound database (http://www.kegg.jp/kegg/compound/). The annotated metabolites were mapped to KEGG pathways (http://www.kegg.jp/kegg/pathway.html) [39, 40].

### Identification of phosphorus level-specific modules and creating a gene interaction network

The regulatory link between genes and other genes is represented by gene connectivity within a module. More connection indicates that the genes in the module have a larger regulatory role and are more likely to develop as core genes. By calculating the KME (module gene-based connectivity) values of genes in the module, the first 20 genes were initially screened as candidate core genes, and then by using Cytascape 3.6.1 [41] (https://cytoscape.org) The Cytonca [42] plug-in calculates the BC (betweenness) value for core gene screening and gene interaction network construction.

### Transcription factor analysis

As molecular switches that regulate the expression of stress-responsive genes, transcription factors (TFs) are essential for the regulation of abiotic stress responses. [43], The protein sequences of the screened modular core genes were submitted to the plant TFDB database [44]. The blast was selected for transcription factor analysis prediction to obtain transcription factor families in each module, which was also submitted to the TAIR Arabidopsis website for functional analysis to further understand the functions of the core genes.

## Supplementary Information

**Additional file 1: Table S1.** Key module genes GO enrichment.

**Additional file 2: Table S2.** Module candidate genes.

**Additional file 3: Table S3.** Top 20 candidate core genes with KME values for key modules.

## Data Availability

The original contributions presented in the study are publicly available. This data can be found here National Center for Biotechnology Information (NCBI) SRA database under accession number a SRP430549. The names of the repository and accession number(s) can be found below: https://www.ncbi.nlm.nih.gov/sra/SRP430549. We hereby declare that the materials used in this study (dianli-1299, dianli-71) were independently selected and bred by Qin Peng’s group at Yunnan Agricultural University and have the right to use them.

## Abbreviations

BC: Betweenness
TFs: Transcription factors
WGCNA: Weighted gene co-expression network analysis
ME: Module eigengene
GS: Gene Significance
MM: Module Membership
KME: Module gene-based connectivity
